# Metastatic Urothelial Cell Carcinoma Presenting as an Isolated Malignancy to the Posterior Fossa: A Case Report and Review on the Literature

**DOI:** 10.7759/cureus.31909

**Published:** 2022-11-26

**Authors:** Louis Reier, Fiona Pudewa, Jordan M Hough, Christina Mao, Javed Siddiqi

**Affiliations:** 1 Neurosurgery, Arrowhead Regional Medical Center, Colton, USA; 2 Neurosurgery, California University of Science and Medicine, Colton, USA; 3 Neurology, St. George's University School of Medicine, Whittier, USA; 4 Neurosurgery, Riverside University Health System Medical Center, Moreno Valley, USA

**Keywords:** posterior fossa, cerebellum, isolated brain metastasis, brain metastasis, bladder cancer, urothelial cell carcinoma

## Abstract

Urothelial cell carcinoma (UCC) of the bladder infrequently metastasizes to the central nervous system (CNS). The incidence worldwide is approximately 1%. The cerebral hemispheres of the anterior and middle cranial fossa are the most common sites of CNS spread, and usually, multiple metastatic lesions are present. Infrequently, metastasis presents as a single solitary metastatic malignancy to the posterior fossa. Here, we present the case of a patient with bladder UCC who presented with a single solitary metastatic malignancy to the cerebellum. The authors discuss the signs, symptoms, mechanism of metastatic spread to the CNS, diagnosis, management, and prognosis of isolated posterior fossa metastasis originating from bladder UCC. We also performed an extensive literature search to identify all cases of metastatic bladder UCC presenting as an isolated malignancy to the posterior fossa in the past 20 years.

## Introduction

Bladder cancer is the sixth most prevalent malignancy in the United States [1]. Urothelial cell carcinoma (UCC) makes up 90% of all types of bladder cancer [1,2]. The literature estimates that distal metastasis occurs in 10%-29% of patients with bladder UCC. The most common sites of distal metastasis, in order of most to least prevalent, are the lymph nodes, liver, peritoneum, lungs, and bones [2,3]. Metastasis to the central nervous system (CNS) can occur but is extremely rare [1]. The first case ever reported was in 1924 [1]. Most studies report the incidence of CNS metastasis from UCC of the bladder to be 1% [4]. However, others estimate anywhere from 0.3% to 8% [5,6].

When bladder UCC metastasis to the CNS occurs, usually, multiple metastatic lesions are present and most commonly involve structures of the anterior and middle cranial fossa (rarely, the posterior fossa) [4,7]. Here, we present the case of a patient with a history of bladder UCC who presented with a single solitary metastatic lesion to the cerebellum.

## Case presentation

A 43-year-old Hispanic male with a past medical history of bladder urothelial cell carcinoma (UCC), initially diagnosed one year ago, with known metastasis to his kidneys and malignant right pleural effusion, presented to our hospital with a chief complaint of headache and feeling off-balance for six days prior to evaluation. He stated that his headaches were bitemporal, debilitating, 10/10 severity, and constant, with no exacerbating or alleviating factors. Associated symptoms included intermittent confusion, cold sweats, dizziness, diaphoresis, nausea, vomiting, and photophobia. On physical examination, he had a Glasgow Coma Scale of 15; the only abnormal finding was bilateral dysmetria more prominent on the right side with finger-to-nose testing. No additional neurological deficits were appreciated.

His bladder UCC treatment began one year ago, with a chemotherapy regimen of gemcitabine and carboplatin, which he completed in four cycles over the span of three months. Upon completion, a follow-up positron emission tomography (PET) scan showed the regression of his bladder cancer, and he was started on avelumab (immunotherapy) maintenance infusions every two weeks. Other pertinent medical history included prior tobacco and alcohol abuse, as well as nephrostomy tubes placed shortly after he was initially diagnosed with UCC one year ago.

Imaging on presentation

**Figure 1:**
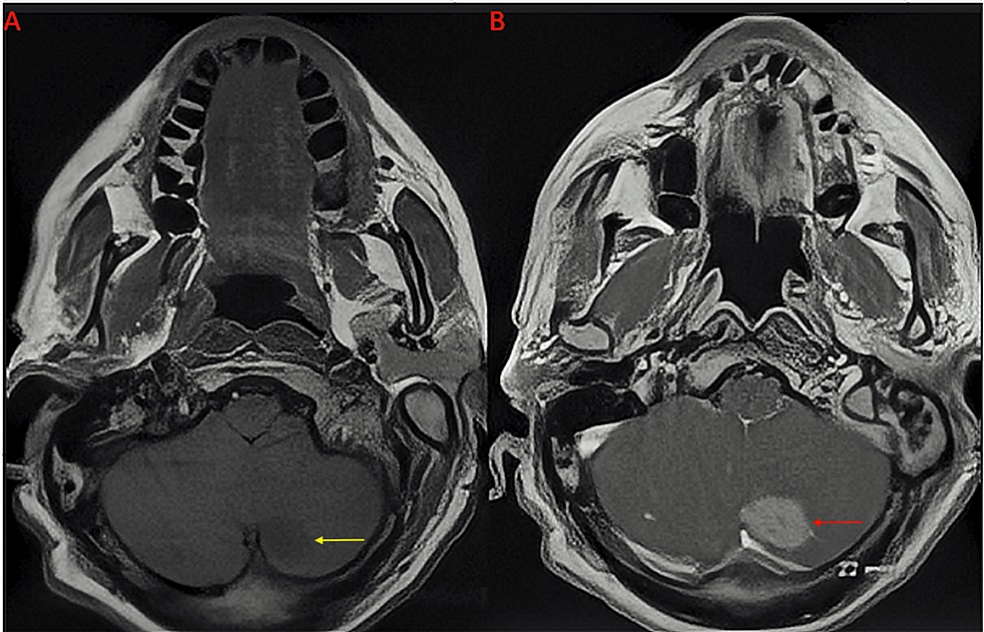
Preoperative MRI of the brain with and without contrast in the axial plane Precontrast (A) and postcontrast (B). The lesion is hypointense on precontrast T1-weighted sequence (yellow arrow) and shows diffuse postcontrast homogenous enhancement (red arrow) MRI: magnetic resonance imaging

**Figure 2:**
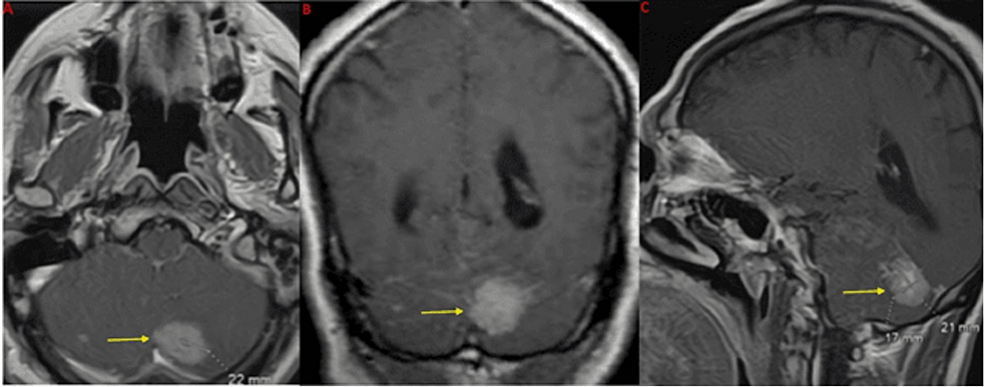
Preoperative MRI of the brain postcontrast sequences in the (A) axial, (B) coronal, and (C) sagittal planes, respectively Yellow arrows point to the postcontrast-enhancing mass MRI: magnetic resonance imaging

Computed tomography (CT) of the head without contrast demonstrated a small hypodensity in the left cerebellar cortex. Follow-up magnetic resonance imaging (MRI) of the brain with and without contrast revealed a single postcontrast homogenously enhancing lesion located posteromedially within the left cerebellar hemisphere with tentorial attachments, measuring 2.2 cm × 2.1 cm × 1.7 cm (as seen in Figures 1-2). There was mild vasogenic edema with associated mass effect and partial effacement of the fourth ventricle; however, hydrocephalus was not present.

CT of the chest, abdomen, and pelvis with and without contrast was also completed and did not show any signs of further metastatic progression when compared to his prior imaging.

Treatment and surgical resection

Decadron was started immediately after the MRI of the brain was reviewed. He was given a 10 mg loading dose, followed by a standing order of 4 mg every six hours (with concurrent proton pump inhibitor and sliding scale insulin). He then consented to a suboccipital craniotomy for the resection of the mass, with the primary goals of surgery being diagnosis and decompression.

**Figure 3:**
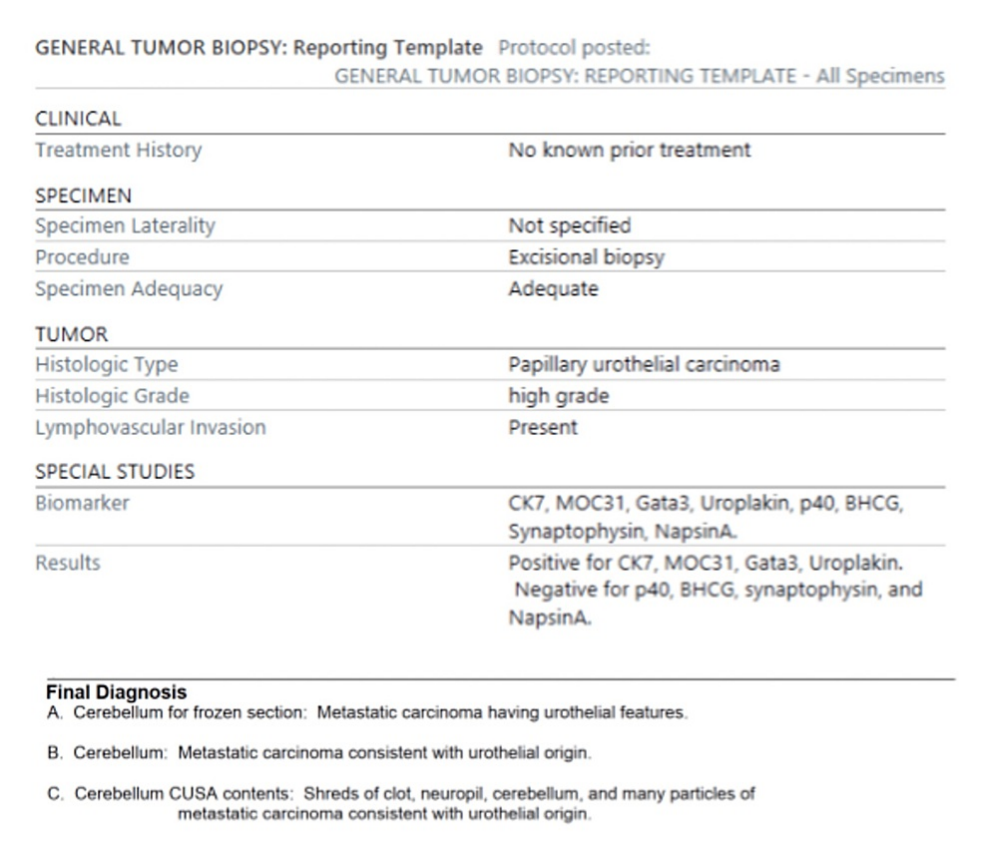
Final pathology report All patient-specific identifiers have been removed BHCG: beta-human chorionic gonadotropin; CK7: cytokeratin 7; CUSA: cavitron ultrasonic surgical aspirator; GATA3: GATA binding protein 3; MOC31: monoclonal antibody 31

The patient was positioned in the Concorde position; a midline incision was made from 2 cm above the inion to the spinous process of the second cervical vertebrae. Dissection continued in the midline avascular plane. At the cranial end, dissection was taken down to the periosteum of the occipital bone at the level of the inion. At the caudal end, dissection was taken down to the top of the posterior arch of C1. Monopolar electrocautery was used to widen the exposure of the occiput laterally. The Stealth navigation probe (Medtronic Minimally Invasive Therapies, Minneapolis, MN) was then used to identify the torcula, transverse sinus, and tumor borders. One burr hole was then placed on each side of the external occipital crest, and the footplate drill was then used to complete the craniotomy. Prior to opening the dura, a micro-Doppler was used to identify the inferior border of the transverse sinus and torcula. A number 11 blade and Woodson were then used to open the dura in a "Y"-shaped fashion. The tumor was immediately visible, and a biopsy was taken from the central portion and sent for a frozen section. Gentle suction and bipolar electrocautery were used to create a plane between normal cerebellar tissue and the mass. Cottonoid patties were placed circumferentially within the brain-tumor interface, and the remainder of the lesion was resected en bloc. Surgicel (Ethicon, Cincinnati, OH) was used to line the resection cavity in proximity to the torcula and transverse sinus, and hemostasis was obtained. No residual tumor was seen with ultrasound (with and without Valsalva). A DuraGen (Integra LifeSciences Corporation, Princeton, NJ) was placed as an inlay inside the native dural leaflets (which were reapproximated with 4-0 Nurolon {Medline Industries, Inc., Northfield, IL} sutures). Tisseel (Baxter Healthcare Corporation, Deerfield, IL) was used overlying the closure, and a single titanium mesh was used for the cranioplasty. The closure was done in a typical multilayer fashion, with the application of staples to approximate the skin edges. No drain was placed. He was extubated in the operating room and immediately followed commands and was moving all extremities with good strength. Pathology resulted in high-grade metastatic papillary urothelial cell carcinoma (full pathology report is shown in Figure 3). Adjuvant radiation therapy and chemotherapeutics were planned following discharge.

Postoperative imaging

**Figure 4:**
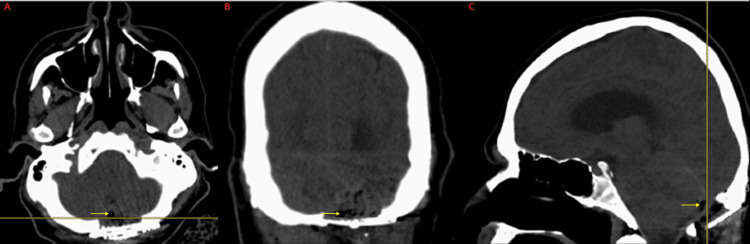
Immediate postoperative CT of the head without contrast in the axial plane (A), coronal plane (B), and sagittal plane (C) A small amount of pneumocephalus is present in the surgical cavity (yellow arrows), which is an expected postoperative finding CT: computed tomography

CT of the head immediately following surgery showed typical postoperative changes (as seen in Figure 4), with a small amount of pneumocephalus in the surgical bed. No hemorrhage, mass effect, or change in ventricle size was seen.

**Figure 5:**
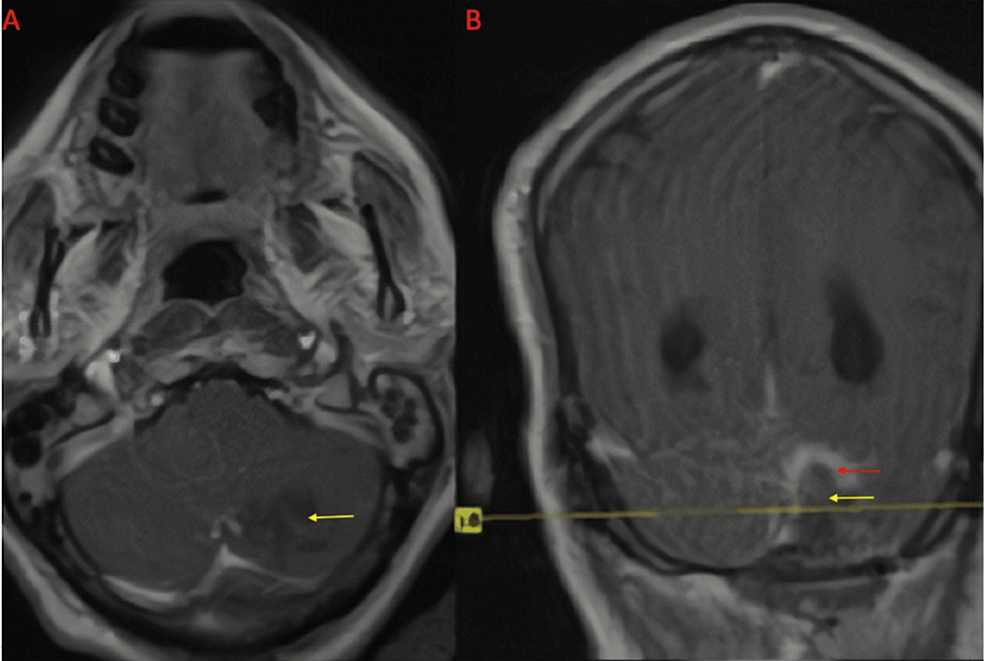
Postoperative MRI of the brain T1-weighted postcontrast imaging in the (A) axial and (B) coronal planes There is interval mass resection at the left cerebellum (yellow arrows). There is persistent enhancement along the surgical margin (red arrow, Figure 5B), which was attributed to hemostatic agents used to fill the tumor cavity during surgery MRI: magnetic resonance imaging

**Figure 6:**
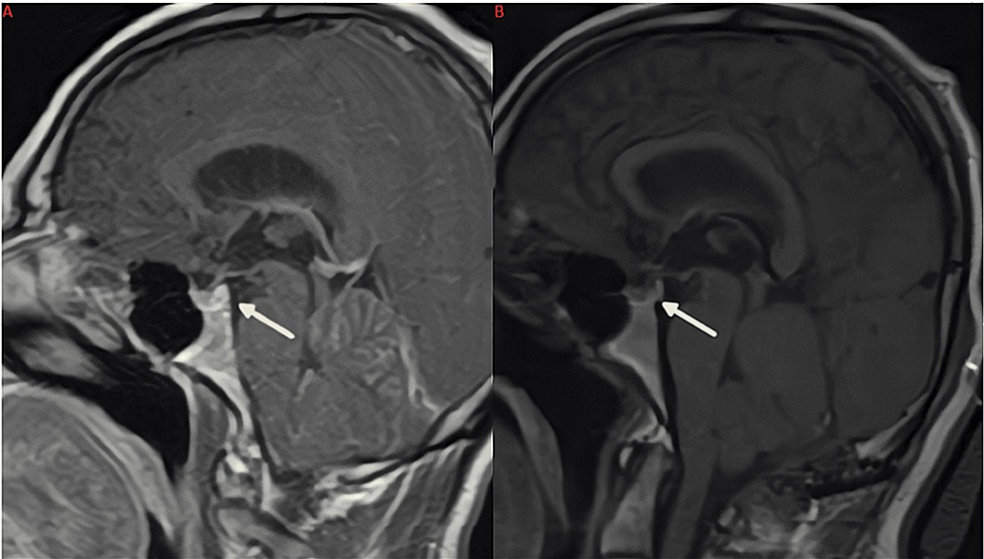
(A) Preoperative MRI (left) is shown for comparison. (B) MRI of the brain postcontrast in the sagittal plane A partially empty sella sign is seen on the postoperative MRI that was not present prior to surgery (yellow arrows pointing to sella). The empty sella sign is a radiographic indicator of hydrocephalus MRI: magnetic resonance imaging

Postoperative MRI of the brain with and without contrast was obtained on postoperative day (POD) 1 and is shown in Figures 5-6. In Figure 5B, persistent enhancement is seen along the superior border of the resection cavity, which was attributed to hemostatic agents used to fill the tumor cavity at the conclusion of surgery. Although not visible in Figure 5 or Figure 6, there is also surrounding edema with partial effacement of the fourth ventricle, as well as slightly enlarged lateral ventricles. In the sagittal plane, a partially empty sella sign is seen (Figure 6B), which was a new finding compared to preoperative MRI (Figure 6A). Although the development of hydrocephalus was a concern radiographically, the patient's examination remained stable at this time.

Postoperative clinical course

In the initial postoperative period, the patient stated that his headaches improved drastically, and no changes were seen on physical examination. However, on postoperative day (POD) 4, his excruciating bitemporal headaches returned, he became agitated and combative, and his mental status declined (alert and oriented to name only while previously alert and oriented to person, place, and time). CT of the head without contrast was ordered and revealed hydrocephalus, with noticeably larger ventricles. An external ventricular drain (EVD) was placed, and the patient's symptoms and mental status immediately returned to baseline. The EVD could not be successfully weaned; thus, on EVD day 10 and POD 14, a programmable ventriculoperitoneal shunt (VPS) was placed. After his VPS was placed, he remained stable from a neurological standpoint. Three days later, he aspirated, and a chest film showed interval development of complete opacification of the right hemithorax, and he went into respiratory failure. Emergent intubation was offered; however, the family declined and requested his code status be changed to do not resuscitate/do not intubate (DNR/DNI), and goals of care were changed to comfort measures. Unfortunately, the patient expired the next morning.

## Discussion

Metastatic brain cancer is the most common type of adult brain tumor, accounting for just over 50% of all brain neoplasms [8]. The most common primary source is lung cancer, followed by breast cancer, kidney cancer (specifically renal cell carcinoma), cancer of the gastrointestinal tract, and melanoma [8]. Urothelial cell carcinoma (UCC) of the bladder is the sixth most common type of adult cancer and rarely the primary source of brain metastasis (the estimated incidence is 1% of all cases of bladder UCC) [5,6]. Cases of UCC with metastasis to the central nervous system (CNS) are most often seen in males, with an average age of 60 at the time of diagnosis [1]. Headache is the most common presenting complaint (as seen in our patient), multiple metastatic lesions are typically present on initial diagnosis, and the most common site of CNS metastasis is the cerebral hemispheres of the anterior and middle cranial fossa (posterior fossa less commonly involved) [1,4,9]. In a retrospective review by Diamantopoulos et al., all cases of metastatic UCC with CNS metastasis treated at their institution between the years 2006 and 2018 were reviewed. Only a total of 20 cases were found. Among these 20 cases, only one case presented with a single metastatic lesion to the cerebellum, exemplifying its rarity [1].

This prompted our extensive literature search in an attempt to identify all reported cases of metastatic UCC presenting with a single solitary malignancy to the posterior fossa in the past 20 years. We found a total of 11 cases reported, 10 from published case reports and one identified from the analysis of an institutional retrospective review article. Table 1 is a summary of all 11 cases ordered by year of publication.

**Table 1:** Summary of cases reported in the past 20 years of urothelial cell carcinoma of the bladder presenting with a single solitary metastatic lesion to the posterior fossa cc: chief complaint; CNS: central nervous system; CT: computed tomography; ED: emergency department; MRI: magnetic resonance imaging; PET: positron emission tomography; UCC: urothelial cell carcinoma

Study	Type	Summary of study and findings
Majcherczyk et al., 2021 [10]	Case report	A 55-year-old male with cc of headaches and cerebellar symptoms starting three weeks ago. CT and MRI revealed a tumor in the cerebellar vermis measuring 2.8 cm × 2.4 cm × 2.2 cm. A metastatic workup found a bladder tumor causing right-sided hydronephrosis. Both cerebellar and bladder tumors were resected, and both histopathologic and immunohistochemical examinations confirmed high-grade muscle-invasive bladder cancer. Received radiotherapy and systemic chemotherapy after surgery. Follow-up information not available.
Diamantopoulos et al., 2020 [1]	Retrospective review	Retrospective review with the purpose of identifying all patients with CNS metastasis secondary to UCC of the bladder treated at their institution between January 2006 and December 2018. Twenty patients were identified. Of these 20 patients, only one had a single metastatic lesion to the posterior fossa.
Worm et al., 2016 [3]	Case report	A 72-year-old female with cc of headaches and cerebellar symptoms starting two months prior to presentation. Medical history was significant for surgical resection of UCC of the bladder two years ago. Pertinent physical examination findings include cerebellar impairment (loss of balance, dysmetria, and dysdiadochokinesia). MRI showed an expansive posterior fossa mass (5 cm × 4 cm) located in the cerebellar vermis. Surgically resected and pathology consistent with metastases from UCC of the bladder. The patient developed intracranial hypertension and had a ventriculoperitoneal shunt (VPS) placed. The patient died 20 months later from systemic complications of extracranial tumor return.
Kelten et al., 2015 [11]	Case report	A 75-year-old male, who was diagnosed with UCC of the bladder and underwent transurethral resection of the tumor, presented 18 months later with confusion, dizziness, vomiting, and slurred speech. Imaging showed a 2.8 cm × 1.9 cm mass lesion within the cerebellum, which was surgically resected. Histopathologic studies and immunohistochemistry panel reported high-grade metastatic UCC. Follow-up information is not available.
Kartha et al., 2015 [12]	Case report	A 59-year-old male presented to the ED with cc of headaches. CT of the head showed a 4 cm × 3 cm × 2 cm mass in the right cerebellar vermis. Underwent surgical resection and pathology consistent with UCC of the bladder. A metastatic workup followed and showed a bladder mass, which was also surgically resected, and pathology was consistent with that of the brain mass. He underwent six weeks of whole-brain radiation treatment once discharged from the hospital. Did not undergo chemotherapy. The patient expired 11 years later due to the recurrence of cancer and the recurrence of brain metastasis involving multiple locations.
Vaa et al., 2014 [7]	Case report	A 65-year-old male with history of UCC of the bladder, diagnosed two years ago and initially treated with cisplatin and gemcitabine. Reoccurred seven months after the completion of chemotherapy and then underwent penectomy, total urethrectomy, and neobladder takedown. Three months following surgery, presented with cc of headaches, lethargy, and cerebellar symptoms (ataxia, unsteadiness, and falls) for the past week. Imaging revealed a single 3.9 cm × 3.4 cm mass within the posterior right cerebellar hemisphere. The patient declined treatment due to an overall poor prognosis, was discharged home with hospice, and expired shortly after.
Gardner and Tan, 2013 [13]	Case report	A 74-year-old male presented with symptoms of urinary tract obstruction and was found to have a bladder mass, and a biopsy showed high-grade papillary UCC. Eleven months after completing chemotherapy, he presented back with cc of fatigue and cerebellar symptoms (unsteady gait, dizziness, disequilibrium, and diplopia). MRI of the brain showed a single isolated mass in the left cerebellar vermis. Underwent surgical resection; pathology was consistent with metastatic UCC. Subsequently treated with whole-brain radiation treatment. The patient did well following radiation treatment for nine months, at which point he developed recurrence and evidence of further metastatic spread. Was then enrolled in hospice, and expired shortly after.
D'Souza et al., 2011 [4]	Case report	A 69-year-old female with a medical history of UCC of the bladder, status post (s/p) surgical resection 12 months ago. Presented with slurred speech, facial droop, and ataxia. CT showed a 4.8 cm right cerebellar mass, with no other distant metastases identified. Treated with surgical debulking followed by whole-brain radiation treatment. The most recent follow-up information available is from 21 months post whole-brain radiation, at which point the patient was doing well with no evidence of recurrence or further metastatic disease.
Perlmutter et al., 2006 [14]	Case report	This case is about a patient who developed isolated cerebellar metastasis from UCC of the bladder. However, the full-text article is not accessible to the public. No further information known.
Kobayashi et al., 2004 [15]	Case report	A 70-year-old male who was nine months status post nephroureterectomy and partial colectomy for UCC of the bladder with invasion into the ascending colon presented with cc of headaches, nausea, and dizziness. CT of the head showed a 4 cm-diameter mass in the left cerebellar hemisphere. Surgically resected; pathology showed poorly differentiated UCC, similar to the original bladder lesion. Patient expired shortly after.
Davies et al., 2003 [16]	Case report	A 56-year-old male presented with cc of ataxia and headaches in the absence of any urologic symptoms. Imaging showed right cerebellar tumor measuring 2.5 cm × 1.4 cm × 0.4 cm. Underwent surgical resection; pathology demonstrating histologic features consistent with high-grade UCC. Following surgery, underwent metastatic workup and PET scan, which showed suspicious lesion in posterior bladder wall; cystoscopy confirmed high-grade UCC (similar to cerebellar pathology). Full-text article not available to public and thus no further information regarding patient follow-up available and overall outcome unknown.

Single solitary lesions intuitively have a better prognosis than multiple lesions; however both ultimately have a poor prognosis [1]. In a retrospective review by Rosenstein et al., mean survival from the time of diagnosis in those with a single solitary lesion was 14 months, compared to three months in those with multiple metastatic lesions [17].

Metastatic spread to the CNS occurs via two different routes: visceral organs and vertebral venous plexus [18]. Visceral organ route is the most common route and refers to typical hematogenous dissemination. Cancer cells first infiltrate the lungs; then, secondary spread to the brain occurs. This method presents more indolently than primary brain metastasis, and death typically occurs prior to the manifestation of neurological symptoms [19]. Metastasis by route of the vertebral venous plexus is less common [18]. The vertebral venous plexus is a giant network of interconnecting valveless veins along the vertebral column, extending from the sacrum all the way to the foramen magnum. Thus, metastasis via this route bypasses the lungs, liver, and other visceral organs traveling directly into the intracranial venous sinus at the level of the foramen magnum [3,18]. This is speculated to be the route of metastasis in most cases of single isolated CNS metastasis secondary to bladder UCC. It is also thought that this route of metastasis is more common in UCC originating from the lower genitourinary tract [18-20].

The gold standard treatment for bladder UCC is cystectomy combined with a chemotherapy regimen consisting of methotrexate, vinblastine, doxorubicin (adriamycin), and cisplatin (commonly referred to as M-VAC). Although these chemotherapeutic agents have been quite successful in treating local UCC and visceral organ spread, they have poor penetrance through the blood-brain barrier. It is proposed that there may be a subpopulation of cancer cells that are able to seed the CNS, survive the chemotherapy effects, and later present with CNS metastasis in the absence of other distal metastasis [2,18].

Finally, although not the primary learning point from this case report, another interesting finding seen was the partial empty sella sign on postoperative MRI (as seen in Figure 5). In the literature, the empty sella sign is either attributed to elevated intracranial pressure (ICP) or reported as an incidental finding [21]. Interestingly, our patient developed this finding prior to ventricle size change or the onset of symptoms. More research should be done on the pathophysiology of empty sella sign in relation to the stages of hydrocephalus development.

## Conclusions

Central nervous system (CNS) metastasis from bladder urothelial cell carcinoma (UCC) continues to be a rare disease entity, occurring in roughly 1% of all patients with metastatic UCC. UCC presenting as a single metastatic lesion to the posterior fossa is seen even less frequently. We identified 11 reported cases over the past 20 years, our case being the 12th. As demonstrated by this case of a small, solitary, brain metastasis of urothelial cell carcinoma, this is a rare but ominous condition with a grave prognosis (despite what appeared as a relatively small intracranial burden of disease).
